# Human NCF1^90H^ Variant Promotes IL-23/IL-17—Dependent Mannan-Induced Psoriasis and Psoriatic Arthritis

**DOI:** 10.3390/antiox12071348

**Published:** 2023-06-27

**Authors:** Yanpeng Li, Zhilei Li, Kutty Selva Nandakumar, Rikard Holmdahl

**Affiliations:** 1 Division of Medical Inflammation Research, Department of Medical Biochemistry and Biophysics, Karolinska Institute, 17177 Stockholm, Sweden; yanpeng.li@ki.se; 2 SMU-KI United Medical Inflammation Center, School of Pharmaceutical Sciences, Southern Medical University, Guangzhou 510515, China; kutty-selva.nandakumar@hh.se; 3 Clinical Pharmacy Division of Pharmacy Department, Southern University of Science and Technology Hospital, Shenzhen 518055, China; lizhilei@sustech-hospital.com; 4 Department of Environmental and Biosciences, School of Business, Innovation, and Sustainability, Halmstad University, 30118 Halmstad, Sweden

**Keywords:** NCF1, ROS, psoriasis, macrophages, IL-23/IL-17 axis, JAK-STAT

## Abstract

Recently, a major single nucleotide variant on the NCF1 gene, leading to an amino acid replacement from arginine to histidine at position 90 (NCF1^R90H^), associated with low production of reactive oxygen species (ROS), was found to be causative for several autoimmune diseases. Psoriasis in the skin (PsO) and psoriatic arthritis (PsA) were induced with mannan by intraperitoneal injection or epicutaneous application, evaluated by visual and histology scoring. Immunostaining was used to identify macrophages, NCF1, and keratinocytes. The population of immune cells was quantified by flow cytometry, gene expression was analyzed by RT-qPCR, and the JAK/STAT signaling pathway was investigated by immunohistochemical staining and western blot. We found that the low ROS responder NCF1^90H^ variant promotes PsO and PsA (the MIP model). The NCF1^90H^-expressing mice had hyperactivated macrophages, expanded keratinocytes, and dramatically increased numbers of γδT17 cells with upregulated IL-17A, IL-23, and TNF-α. In addition, the JAK1/STAT3 signaling pathway was also upregulated in cells in the psoriatic skin tissues of *Ncf1^90H^* mice. To summarize, a defined SNP (*NCF1-339*, also named NCF1^90H^) was found to activate the IL-23/IL-17 axis and JAK-STAT signaling pathways, leading to hyperactivation of macrophages and keratinocytes and causing mouse psoriasis and psoriatic arthritis.

## 1. Introduction

Chronic autoimmune diseases, such as rheumatoid arthritis (RA), systemic lupus erythematosus (SLE), Sjögren’s syndrome (SS), psoriasis vulgaris (PsO), and psoriatic arthritis (PsA), are common in the population, and they are the subject of intense research to understand the underlying genetic and environmental causes [1]. The main problem is the disease complexity caused by interacting factors, which form network effects that are difficult to decipher. Although studies have shown that inflammation-inducing factors, skin damage, and unknown infections can cause or aggravate the disease process, the exact triggers of PsO and PsA remain unknown.

The first single nucleotide polymorphism to be positioned in polygenic autoimmune diseases was the neutrophil cytosol factor (*Ncf1*), encoding a protein (NCF1, alias p47phox) critically involved in the formation of the NADPH oxidase 2 (NOX2) complex, responsible for the induction of reactive oxygen species (ROS), as an essential regulator of several autoimmune-mediated chronic inflammatory disorders. This was originally done by positional cloning of the genetic polymorphism causing autoimmune arthritis in the rats. This effect was reproduced in mice with a spontaneous Ncf1^m1J^ mutation, followed by the identification of a human SNP (replacing arginine with histidine at position 90 in the NCF1 subunit of NADPH oxidase 2 (NOX2) complexes, i.e., *NCF1^R90H^*) [2,3]. An SNP leading to replacement of threonine with methionine (T153M) in rats in the same NCF1 domain was identified, which has a similar functional effect on the NOX2 complex as the subsequently identified human polymorphism (R90H) [4]. Notably, the NCF1 locus, due to copy number variation (CNV), has not been sequenced or included in genome-wide association screens [3].

Directed analysis showed that the *NCF1^R90H^* mutation facilitated the development of more severe SLE, RA, and SS [5,6]. However, no effect of R90H—only an association with CNV—was found in another cohort of RA [3] Similarly, no association was found with R90H in a PsO/PsA cohort, but an association with loci containing other redox-related genes (NOS2) has been identified [7]. Functional analysis in vivo was possible with the help of a spontaneous mutation in the mouse *Ncf1* gene leading to severe deficiency (the *Ncf1^m1J^* mutation) and a conditional *Ncf1* knock-in [8,9,10,11]. A defective *Ncf1* gene led to more severe diseases in all investigated autoimmune models, including RA, SLE, and PsO/PsA [8,11,12]. Using mouse strains with the *Ncf1^m1J^* mutation on the C57BL/6N.Q genetic background, we found that intraperitoneal injection of mannan induced a disease mimicking PsO and PsA [11]. Another mannan-induced psoriasiform inflammation model in back skin was recently developed using mannan mixed with Freund’s incomplete adjuvant, and this model induced a more severe disease than the classically used imiquimod-induced psoriasis (IMQ) model, with better disease-relapsing features and less toxic effects [13].

We and others have established mouse strains with the human R90H amino acid replacing mutation [14,15,16]. Mice with the NCF1^90H^ mutation are more susceptible to lupus. We now show that the NCF1^90H^ mutation promotes the development of severe mannan-induced PsO and PsA, with IL-23/IL-17 associated pathways typical for human psoriasis.

## 2. Materials and Methods

### 2.1. Mice

*Ncf1^90H^* founder mice were generated using CRISPR/Cas9 technology using C57BL/6N ES cells, as described in previous studies. A *MHCII* (*H2q* containing the *A^q^* allele) haplotype was inserted by backcrossing onto the C57BL/6N.Q to establish the wild-type mice BQ.*Ncf1^R90^* (*Ncf1^R90/R90^*, or *Ncf1^R90^*), heterozygous BQ.*Ncf1^R90/90H^*, and homozygous BQ.*Ncf1^90H^* (*Ncf1^90H/90H^*, or *Ncf1^90H^*) and BQ.*Ncf1^m1J^* strains. Mouse strains with the *H2^q^* haplotype (commonly occurring haplotype in wild mice) have increased susceptibility to several autoimmune disorders, including autoimmune arthritis, PsO, and PsA [17]. Genotyping of mice was routinely performed by PCR and agarose gel electrophoresis with primer set (GenBank Accession: NM_010876; *Ncf1^90H^* forward: AACGAGCCGCTGAGAGTGAC, *Ncf1^90H^* reverse: TTCAGGTCATCAGGCCGCAC; wild-type forward: AACGAGCCGCTGAGAGTGGC, wild-type reverse: TTCAGGTCATCAGGCCGCAC). We used 8- to 12-week-old age- and sex-matched littermates or wild-type controls in all the experiments. The animals were housed in individually ventilated polystyrene cages containing enrichments, placed in a climate-controlled environment with a 14 h light/10 h dark cycle, and with standard chow and water given ad libitum. All the experimental procedures were approved by the local ethical committee (Guangzhou, China, permit number: L2020013) and performed following the ARRIVE guidelines [18].

### 2.2. Measurement of ROS

ROS production was quantified as described previously [8]. For intracellular ROS, 1 × 10^5^ cells per well were stimulated with or without phorbol myristate acetate (PMA) ex vivo, and dihydrorhodamine-123 (DHR) fluorescence was used for detection. The samples were measured using LSR Fortessa (BD Biosciences, San Jose, CA, USA) and analyzed in FlowJo software V10. For extracellular ROS measurement, PBMCs isolated by Ficoll-Paque Plus (GE, Chicago, IL, USA), bone marrow cells, and splenocytes were collected, lysed, and washed with PBS and then treated with 100 μL of isoluminol reagent buffer, as previously described [6]. Data were collected using a Biotek plate reader. For ROS measurement in vivo, sedated mice were injected i.p. with 2 mg/kg of LPS (100 μL) for five hours. Subsequently, we used 75 mg/kg of L-012 probe (Wako Chemicals, Neuss, Germany) dissolved in physiological saline (100 μL). A luminescent signal was detected using the IVIS 50 bioluminescent system. Ex vivo imaging was done on dissected organs. We used Living Image software (Xenogen, San Francisco Bay Area, CA, USA) and SPSS 29 (Chicago, IL, USA) for data acquisition and analyses, respectively. Image exposure times were between 5 s and 2 min, depending on the signal strength. Light emission from the region of interest was quantified as photons/second cm^2^ steradian.

### 2.3. Western Blot

Skin samples were obtained from the psoriatic mice ten days after mannan injection or application for analysis. Total proteins from the skin samples extracted using radioimmunoprecipitation assay (RIPA) lysing buffer were used to determine the concentration of proteins using a BCA protein assay kit (Pierce, Rockford, IL, USA). The supernatants from protein lysates were subjected to SDS-polyacrylamide gel electrophoresis (PAGE) (Invitrogen, Waltham, MA, USA) and then transferred to PVDF membranes. The following primary antibodies were used for analysis and incubated at 4 °C overnight with JAK1 (6G4) Rabbit mAb (3344, CST, Danvers, MA, USA), p-JAK1(Tyr1034/1035) (D7N4Z) Rabbit mAb (74129, CST, USA), STAT3 (79D7) Rabbit mAb (4904, CST, USA), or p-STAT3 (Tyr705) (D3A7) XP Rabbit mAb (9145, CST, USA). The signals were detected using anti-rabbit IgG conjugated with HRP as the secondary antibody (CST, Danvers, MA, USA) and an electrochemiluminescence system (ECL, Thermo Fisher Scientific, Waltham, MA, USA). Data were analyzed using ImageJ software v1.54 (Bethesda, MD, USA).

### 2.4. Mannan-Induced Psoriasis (MIP)

A mannan-induced psoriasis model was established as previously described [11]. Twenty mg of mannan from the yeast S. cerevisiae (M7504, Sigma-Aldrich, Burlington, VT, USA) was dissolved in 200 μL of PBS and injected i.p. at day 0. Mice were scored daily in the peripheral joints: one point for each swollen and red toe or joint and five points for a swollen ankle, adding up to a maximum score of 60 points per mouse [19]. The severity and incidence of the Ps-like skin manifestations were monitored on a scale ranging from 1 to 3 per mouse based on the severity of skin scaling on the paws and ears: 1, mild skin peeling; 2, moderate skin peeling; and 3, heavy skin peeling with some hair loss.

A mannan-induced psoriasis-like model of the skin was established [13] recently. The area of 2 cm × 3.5 cm was shaved at the back of mice, and 5 mg of mannan (100 μL/day) mixed with incomplete Freund’s adjuvant (IFA, Sigma-Aldrich, Burlington, VT, USA) in a ratio of 1:1 was applied on the back skin for three consecutive days and scored for nine days. Psoriasis area severity index (PASI) scores were given based on redness (0–4), scales (0–4), and thickness (0–4), with a total score of 12, using following the criteria: 0, none; 1, slight; 2, moderate; 3, severe and 4, apparent signs. The increase in skin thickness was measured using an Ozaki digital caliper (Neill-Lavielle, Bowling Green, KY, USA).

### 2.5. Histology

For H&E staining, skin tissues were collected on day 10, fixed with 4% paraformaldehyde for 48 h, and embedded in paraffin. Skin sections (3.5 μm) were cut and stained by the Hematoxylin and Eosin Staining Kit (Beyotime, Shanghai, China). The slides were then scanned using an inverted microscope (Nikon, Japan), and the skin samples were assessed using the criteria of a histopathological scoring system according to a previously published protocol [20]. For immunohistochemical (IHC) staining, skin sections (3.5 μm) were cut and stained with NCF1 (p47phox) mouse antibody (D-10) (Santa Cruz Biotechnology, Santa Cruz, CA, USA), F4/80 (D2S9R) XP rabbit mAb (70076, CST, USA), or p-STAT3 (Tyr705) (D3A7) XP Rabbit mAb specific rabbit antibodies (9145, CST, USA), respectively. For immunofluorescent staining, skin sections (3.5 μm) were cut and stained with NCF1 mouse antibody (D-10), F4/80 (D2S9R) XP rabbit mAb (70076, CST, USA), and rabbit recombinant monoclonal (EPR17882) Keratin 12/K12 antibody (ab185627, Abcam, Waltham, MA, USA). Goat anti-mouse IgG (H+L) Alexa Fluor™ 488 antibodies or goat anti-rabbit IgG (H+L) Alexa Fluor™ 594 antibodies (Invitrogen, Thermo Fisher Scientific, Waltham, MA, USA) were used as secondary reagents, followed by counterstaining with 4′, 6-diamidino-2-phenylindole DAPI (Vector, Newark, CA, USA), and dried for 30 min before scanning under a confocal microscope LSM880 with Airyscan (CarlZeiss, Oberkochen, Germany). IHC staining results were derived based on staining intensity (4 scores of 0 to 3+) and the positive rate as the predominant parameters, according to the protocol described previously [21]. The staining intensity (negative, low, moderate, and high) and percentage of staining were assessed, and five pathologists calculated a final score per sample. The total proportion of cells staining positively at any intensity was scored as 0 (no cell staining), 1 (when 1% to 25% cells stained; weak staining), 2 (when 26% to 50% cells stained; moderate staining), and 3 (when >75% cells stained; intense staining).

### 2.6. Quantitative Real-Time PCR

After carbon dioxide asphyxiation, psoriatic skin tissues were obtained from age- and sex-matched mutated and wild-type mice. Total RNA was isolated using TRIzol Reagent (Invitrogen, Thermo Fisher Scientific, Waltham, MA, USA), and complementary DNA (cDNA) was synthesized using the First Strand cDNA Synthesis Kit (Thermo Fisher Scientific, Waltham, MA, USA). We evaluated the gene expression by RT-qPCR using Agilent Strata gene Mx3005P with FastStart Universal SYBR Green Master (Roche, Basel, Switzerland) to assess the gene expression. The relative gene expression normalized by β-actin was calculated with the 2^−∆∆CT^ method. The primers used are described in Table S1.

### 2.7. Flow Cytometry

Flow cytometric analyses were performed as described [19]. Briefly, spleen and lymph nodes were sampled, red blood cells were lysed, Fc-receptors were blocked, and surface antigens were stained with fluorescence-labeled antibodies. To stain immune cells, CD11b-FITC, Ly6C-BV605, Ly6G-APC, F4/80-PE, CD11c-PE, B220-Fluor 450, CD317 (BST2, PDCA-1)-APC, CD45-HV500, and FVS780-APC-Cy7 antibodies were used. Antibodies including FVS780-APC-Cy7, CD45.2-APC, CD3-FITC, CD4-PE Cy7, and TCRγ/δ-BV421 were used to evaluate the expression of extracellular surface molecule staining. For intracellular cytokine staining, IFN-γ-BV510 and IL-17A-PE antibodies were used for staining after fixation and permeabilization with Cytofix/Cytoperm solution (BD, Franklin Lakes, NJ, USA). The samples were measured using LSR Fortessa (BD Biosciences, Franklin Lakes, NJ, USA) and analyzed using the FlowJo software V10 (TreeStar, Ashland, OR, USA).

### 2.8. Statistical Analysis

Results are expressed as mean ± SEM. Clinical arthritis and histology scores were analyzed using the Mann–Whitney U-test. The statistical significance of other data was calculated with the One-Way ANOVA combined with Dunn’s multiple comparison tests to compare various groups using GraphPad Prism Software 9.4.1 (GraphPad Software, San Diego, CA, USA). A *p*-value < 0.05 was considered statistically significant.

## 3. Results

### 3.1. Ncf1^90H^ Mice Express an Intact NOX2 Complex with Deficiency of ROS

**Figure 1:**
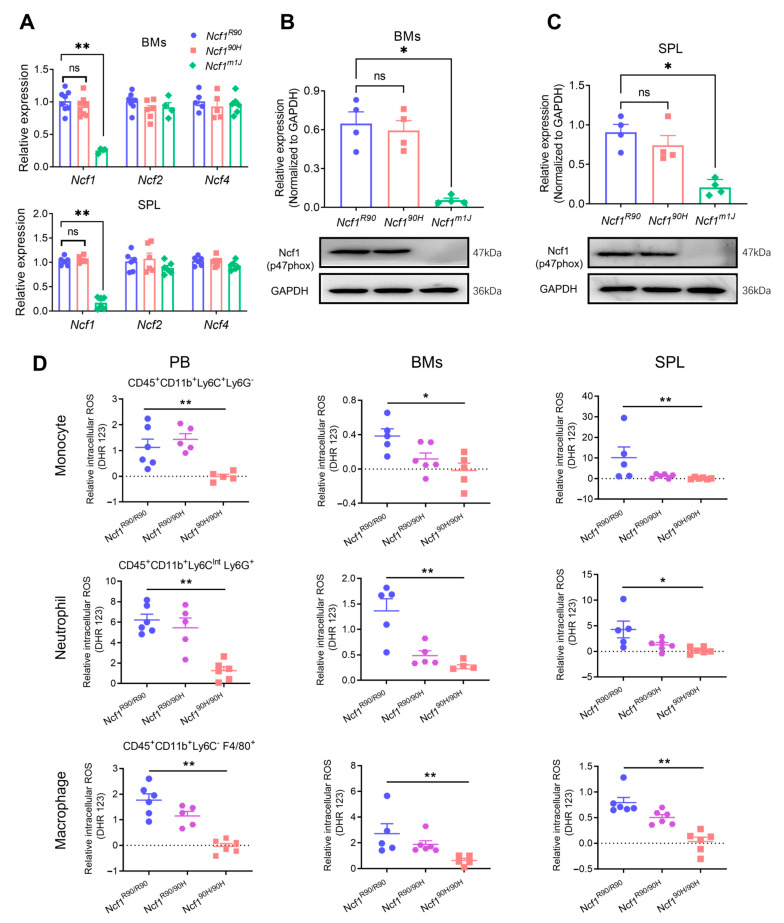
NCF1^90H^ variant decreases intracellular ROS production without altering *Ncf1* expression. (**A**) *Ncf1*, *Ncf2*, and *Ncf4* mRNA. (**B**,**C**) NCF1 protein expression in the bone marrow cells (BMs) and splenocytes of Ncf1^90H^ mice (n = 4–6), quantified by RT-qPCR and Western blot, respectively. (**D**) Relative intracellular PMA-induced ROS levels in monocytes (CD45^+^ CD11b^+^ Ly6C^+^ Ly6G^−^), neutrophils (CD45^+^ CD11b^+^ Ly6C^Int^ Ly6G^+^), and macrophages (CD45^+^ CD11b^+^ Ly6C^−^ F4/80^+^) from peripheral blood, bone marrow, and spleen tissues of homozygous Ncf1^90H/90H^ mice (n = 5–6). Ncf1^R90/R90^ or Ncf1^R90^ denotes wild-type littermates. Ncf1^R90/90H^ and Ncf1^90H/90H^ (also Ncf1^90H^) denote heterozygous and homozygous mice, respectively. Ncf1^m1J^ denotes the negative control. Two independent experiments were operated with a similar trend. The data are shown as mean ± SEM. * *p* < 0.05; ** *p* < 0.01; ns: no significance. PB: peripheral blood; BMs: bone marrow cells; SPL: spleen.

**Figure 2:**
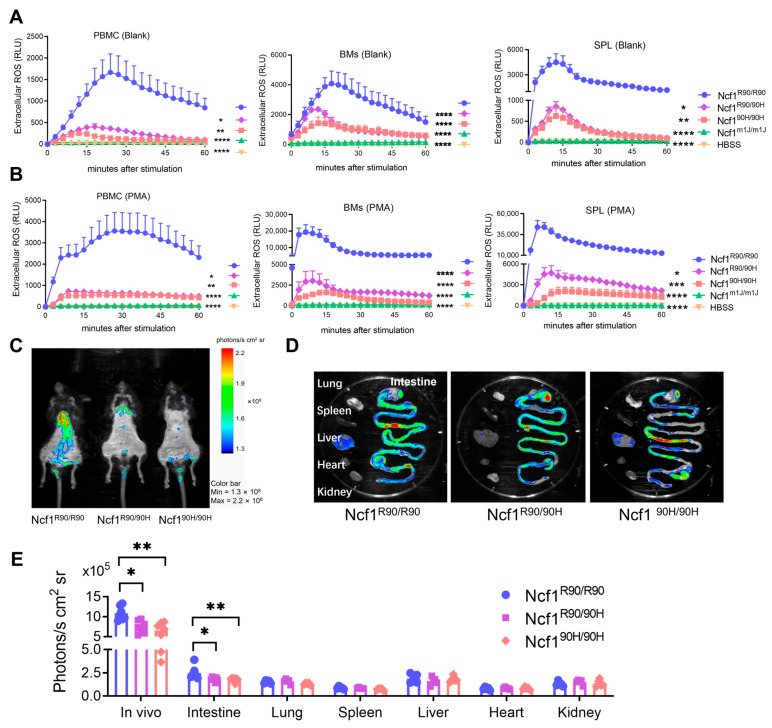
NCF1^90H^ variant decreases extracellular ROS and in vivo ROS production. (**A**,**B**) Extracellular ROS in PBMCs, BM cells, and splenocytes with and without PMA stimulation (n = 5). Hank’s buffer without cells was used as the control. (**C**,**D**) Decreased ROS in vivo and the dissected organs by ex vivo imaging, injected with L-012 probe after LPS stimulation for five hours. (**E**) Quantification of in vivo generated ROS (n = 5–7). Data shown are average luminescence units, and error bars indicate SEM. The pseudo colors represent photons/s cm^2^ sr. Two independent experiments showed a similar trend. The data are shown as mean ± SEM. * *p* < 0.05; ** *p* < 0.01; *** *p* < 0.001; **** *p* < 0.0001. PBMC: peripheral blood mononuclear cell; BMs: bone marrow cells; SPL: spleen.

To investigate the role of the human NCF1^90H^ variant in mouse psoriasis in a NOX2-derived ROS-dependent way, we knocked in the R90H variant in the Ncf1 gene by CRISPR/Cas9 in B6N ES cells and backcrossed to C57BL/6N.Q mice (Figure S1A–D). To address whether the NCF1^90H^ variant influences the expression of cytosolic NOX2 subunits, we examined NCF1 protein and *Ncf1* mRNA expressions in the bone marrow and the spleen of BQ.Ncf1^90H/90H^ mice. We found an intact mRNA expression of *Ncf1*, *Ncf2*, and *Ncf4* in the bone marrow and spleen of Ncf1^90H/90H^ mice and wild-type (Ncf1^R90/R90^) mice (Figure 1A). *Ncf1^90H^* mutated mice expressed intact NCF1 protein in the bone marrow and spleen, in contrast to mice with the *Ncf1^m1J^* mutation. (Figure 1B,C). To investigate the ROS production in immune cells due to the NCF1^90H^ mutation, we measured and found decreased intracellular ROS in monocytes (CD45^+^ CD11b^+^ Ly6C^+^ Ly6G^−^), neutrophils (CD45^+^ CD11b^+^ Ly6C^Int^ Ly6G^+^), and macrophages (CD45^+^ CD11b^+^ Ly6C^−^ F4/80^+^) from the peripheral blood, bone marrow, and the spleen of Ncf1^90H/90H^ mice (Figure 1D and Figure S2). Even a more pronounced decrease in the extracellular ROS was observed within peripheral blood mononuclear cells, bone marrow cells and splenocytes in both blank and PMA-stimulated groups (Figure 2A,B). We found decreased ROS in vivo, in the intestine, of both homozygous (Ncf1^90H/90H^) and heterozygous (Ncf1^R90/90H^) mice, even after LPS stimulation, when compared with wild-type Ncf1^R90/R90^ mice (Figure 2C–E). We conclude that the human NCF1^90H^ variant decreased ROS generation in mice without affecting NOX2 complex protein levels.

### 3.2. Ncf1^90H^ Mice Develop More Severe MIP

**Figure 3:**
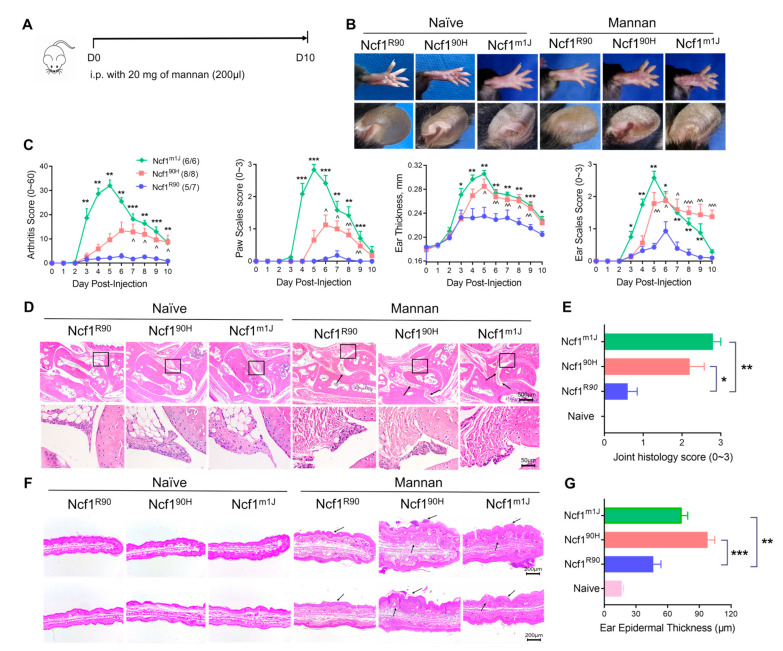
NCF1^90H^ variant aggravates mannan-induced PsA. Characterization of the disease in BQ.Ncf1^R90^, BQ.Ncf1^90H^, and BQ.Ncf1^m1J^ mice induced by mannan, with naive BQ mice as negative controls. (**A**) Timeline of mannan-induced PsA by intraperitoneal injection. (**B**) Representative pictures of psoriatic and arthritic paws and psoriatic ears. (**C**) Arthritis, and paw scale, ear thickness, and ear scale scores. (**D**) HE stain of naïve and inflamed joints. Scale bar = 500 µm or 50 µm. The square indicates synovial hyperplasia and pannus. The arrows indicate inflammatory infiltrates. (**E**) Joint histology scores (n = 6–8). (**F**) HE stain of naïve and inflamed ear tissues. The samples were collected from different parts of the ears, including the edge of the ear (above) and the middle ear (below) in duplicates. Scale bar = 200 μm. The arrows indicate infiltration of inflammatory cells, intracorneal pustules, focal parakeratosis, and intracorneal pustules in ear tissues. (**G**) Ear epidermal thickness (n = 6–8). The data are shown as mean ± SEM. * *p* < 0.05; ** *p* < 0.01; *** *p* < 0.001; ^ *p* < 0.05; ^^ *p* < 0.01; ^^^ *p* < 0.001.

**Figure 4:**
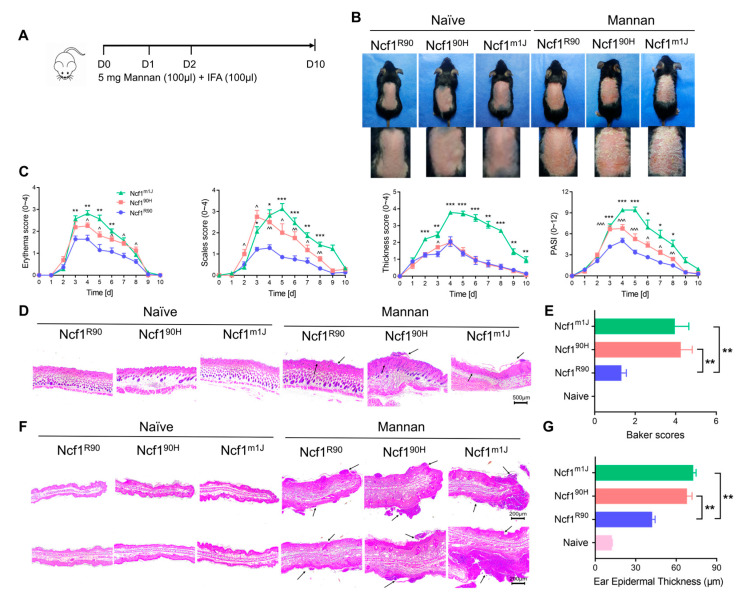
NCF1^90H^ variant aggravates mannan-induced PsO. (**A**) Timeline of mannan-induced PsO by epicutaneous application. (**B**) Representative psoriatic skins of mice on day 4. (**C**) Increased symptoms of erythema, scales, thicker skin, and psoriasis area severity index (PASI) score in Ncf1^90H^ mice (n = 8). (**D**) HE-stained ear skin sections on day 10 (n = 4–6). Scale bar = 500 μm. (**E**) Baker scores. (**F**) HE stain of ear sections (n = 6). Scale bar = 200 μm. (**G**) Ear epidermal thickness. The arrows indicate infiltration of immune cells, and proliferation of keratinocytes. The data are shown as mean ± SEM. * *p* < 0.05; ** *p* < 0.01; *** *p* < 0.001; ^ *p* < 0.05; ^^ *p* < 0.01; ^^^ *p* < 0.001.

To investigate the role of the NCF1^90H^ variant in mouse psoriasis, we induced signs of PsA by injecting intraperitoneally with mannan following the MIP protocol [11]. We found that mice with the NCF1^90H^ variant developed more severe arthritis and psoriasis/dermatitis, observed as swelling of the paws, erythema, scaling of the skin, and thickening and scaling of the ears (Figure 3A,B). We observed increased arthritis and skin scale scores on the paws and increased thickness and scale scores on the ears of mannan-injected Ncf1^90H^ mice and Ncf^m1J^ mice, compared with wild-type Ncf1^R90^ mice (Figure 3C). We also observed more synovial hyperplasia and inflammatory infiltrates in the joints of arthritic paws (Figure 3D). The ear tissue showed more infiltration of inflammatory cells, intracorneal pustules, focal parakeratosis, and intracorneal pustules in mannan-injected Ncf1^90H^ mice and Ncf1^m1J^ mice, as compared with wild-type Ncf1^R90^ or naïve mice of the different genotypes (Figure 3F). The observations were confirmed by joint histopathology scores and ear epidermal thickness (Figure 3E,G). To test whether local exposure to mannan leads to psoriasis-like inflammation in Ncf1^90H^ mice, we applied mannan on the back skins of the mice to induce psoriasis-like inflammation (Figure 4A). We found increased signs of erythema, scaling, and thicker skin on the back of the mice. The PASI scores were enhanced in Ncf1^90H^ mice compared to wild-type Ncf1^R90^ mice, with similar scores as in the positive control Ncf1^m1J^ mice (Figure 4B,C). Histology staining of back skin tissues showed increased infiltration of immune cells, proliferation of keratinocytes, and Baker scores (Figure 4D,E). The ear skin showed increased infiltration of immune cells, proliferation of keratinocytes, and epidermal thickness (Figure 4F,G). Taken together, the NCF1^90H^ variant enhances mannan-induced psoriatic arthritis and psoriasis-like skin lesions.

### 3.3. NCF1^90H^ Variant Promotes the Expansion of Macrophages and Keratinocytes

**Figure 5:**
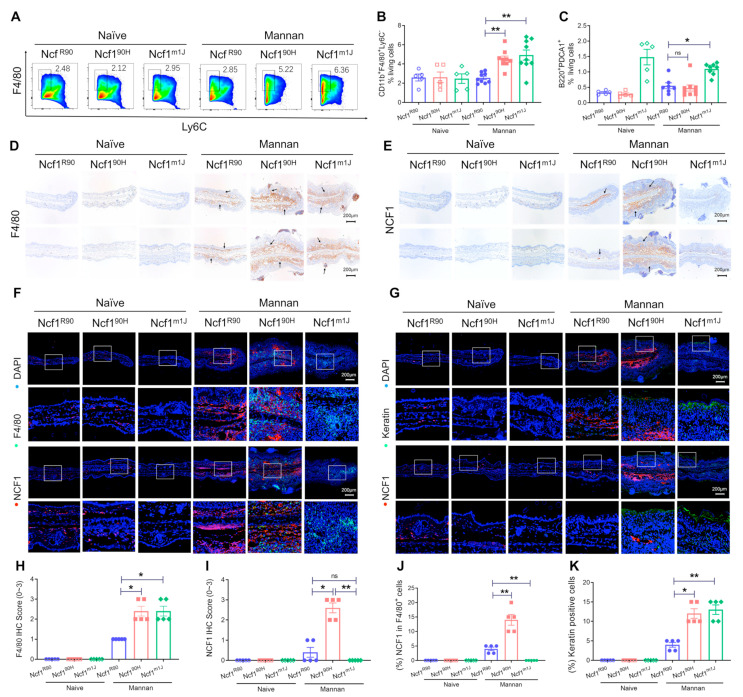
Ncf1^90H^ mice show expansion of macrophages and keratinocytes with NCF1 expression in mannan-induced PsA. (**A**) Gating strategy for macrophages (CD11b^+^ F4/80^+^ Ly6C^−^) in the spleens by flow cytometry analysis on day ten after intraperitoneal injection of mannan. (**B**,**C**) Statistics of macrophage and pDCs (CD45^+^ CD11C^−^ Ly6C^+^ B220^+^ PDCA1^+^) population in the spleen (n = 5–8). (**D**,**E**) Immunohistochemical staining of macrophages (F4/80^+^) and NCF1 proteins in the ears of mannan-injected Ncf1^90H^ mice. Mannan-injected Ncf1^m1J^ mice were used as a positive control, and wild-type Ncf1^R90^ mice were used as a negative control. Scale bar = 200 μm. (**F**) Co-staining of NCF1 and macrophages (F4/80^+^) in ear skin tissue. (**G**) Co-staining of NCF1 and keratinocytes in skin tissue. Scale bar = 200 μm. (**H**) Statistics on IHC scores of macrophages (F4/80^+^) and (**I**) NCF1 in the skin tissues. (**J**) Statistics on expression of NCF1 in macrophages (F4/80^+^) and (**K**) the ratio of positive keratinocytes in the skin tissues. The data are shown as mean ± SEM (n = 5). * *p* < 0.05; ** *p* < 0.01; ns: no significance.

To investigate the immune response after mannan injection, we found an expansion of macrophages (CD11b^+^ F4/80^+^ Ly6C^−^) but not of pDCs (CD45^+^ CD11cCD11b^+^ F4/80^+^ Ly6C^−^ Ly6C^+^ B220^+^ PDCA1^+^) in the spleen of Ncf1^90H^ mice, when compared with wild-type (Ncf1^R90^) mice (Figure 5A,B and Figure S3). However, we noted an expansion of pDCs in mannan-injected Ncf1^m1J^ mice (Figure 5C). There were no noticeable effects on the ratio of neutrophils (CD11b^+^ Ly6G^+^ Ly6C^int/−^) or monocytes (CD11b^+^ Ly6C^+^ Ly6G^−^) in the spleen of Ncf1^90H^ or Ncf1^m1J^ mice (Figure S4). Immunohistochemical staining of the inflamed skins also showed an expansion of macrophages (F4/80^+^) and NCF1-expressing cells in the Ncf1^90H^ mice, compared to wild-type Ncf1^R90^ mice. In contrast, naïve mice without injection of mannan had negligible or very few macrophages or NCF1-expressing cells (Figure 5D,E,H,I), which was also confirmed by co-immunofluorescent staining (Figure 5F,J). In addition, a profound expansion of keratinocytes was seen in Ncf1^90H^ and Ncf1^m1J^ mice after injection of mannan (Figure 5G,K). In the Ncf1^m1J^ mice, we found increased numbers of macrophages and keratinocytes (Figure 5F,G,I,J). Likewise, in both Ncf1^90H^ and Ncf1^m1J^ mice, we noted an expansion of macrophages (F4/80^+^) in the joint tissues from mannan-induced psoriatic arthritis (Figure S5).

### 3.4. NCF1^90H^ Variant Mediates MIP through the IL-23/IL-17 Axis

**Figure 6:**
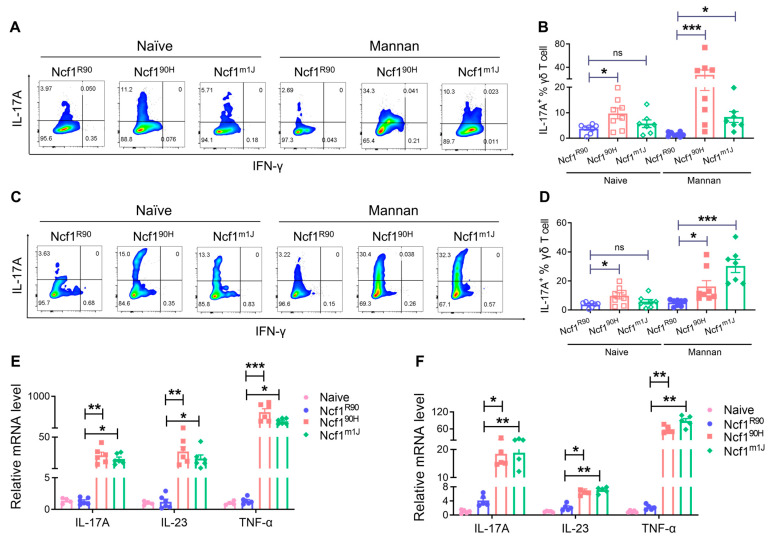
NCF1^90H^ variant mediates IL-23/IL-17 axis in mannan-induced PsA and PsO. (**A**) Gating of γδT17 cells (IL-17A^+^ cells from CD3^+^ TCR^+^ cells) in the lymph nodes by flow cytometry analysis after intraperitoneal injection of mannan. (**B**) Statistics (n = 5–8). (**C**) Gating of γδT17 cells after epicutaneous application of mannan. (**D**) Statistics (n = 5–8). The mRNA expression of IL-17A, IL-23, and TNF-α in the skin tissues by (**E**) intraperitoneal injection and (**F**) epicutaneous application of mannan on the back skin (n = 6). All the samples were collected on day 10. The data are shown as mean ± SEM. * *p* < 0.05; ** *p* < 0.01; *** *p* < 0.001; ns: no significance.

In particular, IL-17 and IL-23 are believed to be the dominant cytokines driving the aberrant activity of T cells and keratinocytes in psoriasis [21,22]. The IL-23/IL-17 axis is important in both human psoriasis and in the MIP model [11,23]. To confirm this, we phenotyped Th cells (CD3^+^ CD4^+^), Th1 cells (CD3^+^ CD4^+^ IFN-γ^+^), Th17 cells (CD3^+^ CD4^+^ IL-17A^+^), and γδT17 cells (CD3^+^ TCR^+^ IL-17A^+^) in lymph nodes from mannan-induced Ncf1^90H^ mice. We found an increased population of γδT17 cells even in naive Ncf1^90H^ mice and a dramatically increased population of γδT17 cells after induction of mannan. The γδT17 cell population expanded after intraperitoneal injection (Figure 6A,B and Figure S6A) and by epicutaneous application of mannan (Figure 6C,D and Figure S6B). We also found an increased population of Th17 cells in Ncf1^90H^ mice only after intraperitoneal injection of mannan, with no changes in the number of Th or Th1 cells after induction of mannan by IP or application on the back skins (Figure S7A,B). The mRNA levels of IL-17A, IL-23, and TNF-α in the skin tissues were increased in Ncf1^90H^ mice by intraperitoneal injection (Figure 6E) as well as by epicutaneous application of mannan (Figure 6F), similar to Ncf1^m1J^ mice, when compared with wild-type (Ncf1^R90^) mice. In conclusion, the NCF1^90H^ variant allows an increased population of γδT17 and Th17 cells, with highly upregulated IL-17A, IL-23, and TNF-α, in the psoriatic skin tissues.

### 3.5. NCF1^90H^ Variant Upregulates JAK1/STAT3 Pathway in MIP

**Figure 7:**
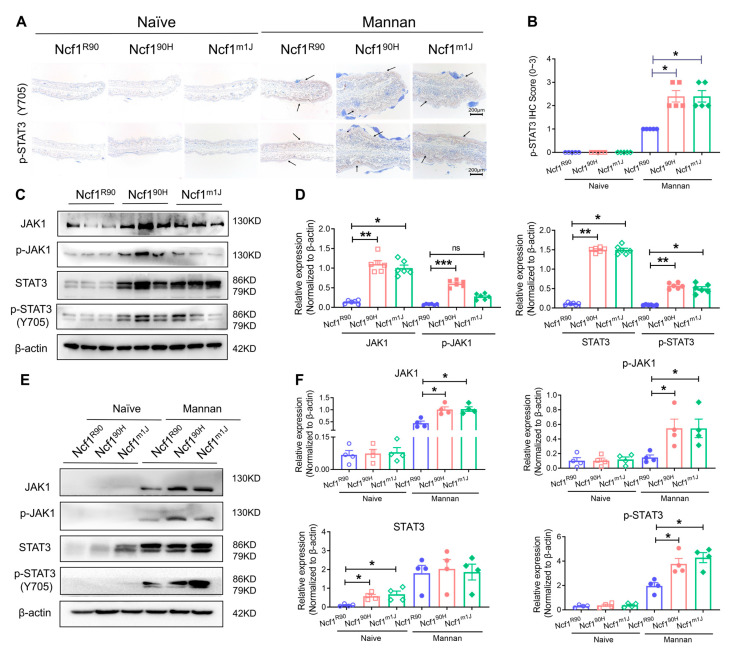
NCF1^90H^ variant upregulates JAK1/STAT3 pathway in mannan-induced PsA and PsO. (**A**) Immunohistochemical staining of p-STAT3 in the ear skin tissues. Scale bar = 200 μm. The arrows indicate the area of positive staining. (**B**) Statistics (n = 5). (**C**) Expression of JAK1/p-JAK1 and STAT3/p-STAT3 proteins in psoriatic skins after intraperitoneal injection of mannan. (**D**) Statistics of Western blot. Immunoblot shows one of the two experiments, with similar results (n = 3 in each experiment) and relative expression with n = 6. (**E**) Expression of JAK1/STAT3 proteins in psoriatic skins after epicutaneous application of mannan on the back skin of mice. (**F**) Statistics of western blot (n = 4). The data are shown as mean ± SEM. * *p* < 0.05; ** *p* < 0.01; *** *p* < 0.001; ns: no significance.

The IL-23/IL-17 axis is known to be highly expressed in psoriatic skin lesions via the JAK/STAT signaling pathway, and JAK inhibitors have been used to treat psoriasis lesions [23,24]. We found high expression of p-STAT3 in the skin tissue of Ncf1^90H^ mice after injection of mannan (Figure 7A,B). An activated expression of JAK1 and phosphorylated JAK1 and STAT3 and phosphorylated STAT3 within the skin tissues of Ncf1^90H^ mice after intraperitoneal injection of mannan was observed (Figure 7C,D). Most importantly, STAT3 expression was upregulated in the naïve skin tissues from Ncf1^90H^ mice and Ncf1^m1J^ mice. In contrast, the expression of JAK1, phosphorylated JAK1, and phosphorylated STAT3 were overexpressed in psoriatic skin tissues of Ncf1^90H^ mice after epicutaneous application of mannan, similar to Ncf1^m1J^ mice, when compared with wild-type (Ncf1^R90^) mice (Figure 7E,F, Figures S4B and S8). Therefore, we conclude that the NCF1^90H^ variant enhances mannan-induced psoriasis in association with an upregulation of the JAK1-STAT3 signaling pathway.

## 4. Discussion

The susceptibility to psoriasis-like inflammation induced by mannan in mice is regulated by ROS from the NCF1/NOX2 complex [11]. We now show that the human *NCF1* polymorphic SNP, replacing arginine with histidine at position 90 (NCF1^R90H^), leads to enhanced susceptibility to MIP. The NCF1^90H^ expressing mice, with lower capacity to induce ROS, develop MIP through activated macrophages and γδ T17/Th17 cells, and are associated with an upregulated STAT3 already in naïve skins, and upregulated JAK1, p-JAK1, and p-STAT3, with highly upregulated IL-17A, IL-23, and TNF-α, in psoriatic skin.

Psoriasis is an immune-mediated and chronic inflammatory skin disease characterized by an excessive immune-mediated inflammatory cell infiltration, leading to hyperproliferation of keratinocytes [25]. Dendritic cells and macrophages release IL-12 and IL-23, which activates T-cells to produce additional cytokines, such as IL-17, IL-22, interferon-gamma (IFN-γ), and TNF-α [26]. Psoriasis pathogenesis involves adaptive and innate T cells, neutrophils, macrophages, and keratinocytes [22,27]. Proinflammatory M1 macrophages contribute to psoriasis development, especially during the early phase, by producing TNF-α [28,29]. Using murine models, it has been shown that macrophage depletion improves psoriasis-like inflammation and reduces proinflammatory Th1 cytokines to normal levels [30].

In the present study, we used not only the MIP model, induced by intraperitoneal injection of mannan, but also local injection of mannan, which could induce increased relapse in the disease. In addition, we also used the earlier described model with local skin application of imiquimod (IMQ), a ligand for Toll-like receptors 7/8, known to induce and exacerbate IL-23/IL-17 axis-dependent PsO in mice [31]. IL-23 stimulate dermal γδ T cells to produce IL-17 and express the IL-23 receptor and transcriptional factor RORγt [32]. We proved that the human Ncf1 polymorphism regulates these models and could activate macrophages and keratinocytes by enhancing the production of TNF-α and IL-23, which triggers IL-17A secretion from γδ T cells, resulting in patchy PsO-like inflammation.

In similarity with human PsA/PsO, the JAK-STAT signaling pathway is activated in mannan-induced psoriatic skin tissues due to NOX2-derived ROS-mediated functions. It is known that multiple cytokines are highly expressed in psoriatic skin lesions, dependent on the JAK/STAT signaling pathway, including IL-19, IL-20, IL-22, and IL-23 [33,34]. Janus kinase inhibitors block the intracellular cytokine-mediated signaling via the JAK-STAT pathway, and they are emerging therapeutics for treating mouse models and psoriasis patients [16,35,36]. Compounds with anti-inflammatory activities protect from psoriasis by regulating intracellular ROS and activation of NF-κB, JAK/STAT, and PI3/Akt signaling cascades, as well as immune responses [37].

The environmental causes of the disease in humans are unknown, but it is likely that an environmental challenge could comprise agents operating as adjuvants of the immune system. One such factor could be mannan, to which we are widely exposed through lungs, gastrointestinal tract, and skin. Mannan is a natural ligand for many C-type lectin receptors (CLRs) expressed on inflammatory cells and known to be a major trigger of IL-17 pathways [11,17]. These CLRs could be both inflammatory and anti-inflammatory, partially dependent on their ability to activate the NOX2 complex. We recently found that polymorphisms of several CLR genes are essential for the susceptibility of autoimmune diseases and control the regulation of the immune response [38]. Activation of many CLRs by selected endogenous or exogenous stimuli, for example, polysaccharides such as mannan, leads to activation of the NOX2 complex, which will regulate the immune system.

Another gene suggested to be associated with PsO and PsA, which is also essential in MIP, is *NOS2* [39], determining the production of NO by macrophages, which in autoimmune models interacts with the regulatory effect of NCF1 [19]. We recently showed that NOS2, and thereby NO production, interacts with NCF1 and the NOX2 complex [40]. Besides the strong influence of the MHC region, a locus with the inducible NOS2 gene is associated with PsO/PsA susceptibility, and the NOS2 locus regulates the exposure of various ROS [19]. Thus, genetic evidence argues for the involvement of non-classical MHC genes involved in innate immune responses regulated by RNS/ROS-mediated functions. Dependent on the immune system, mannan-exposed mice could either develop a disease mimicking RA or mimicking PsO/PsA. Mice with a mutation in *Ncf1* leading to a functionally deficient NCF1, but not the mutation of NCF4, regulate MIP [41], indicating that the NOX2 complex regulates psoriasis and autoimmune arthritis differentially. *NCF1^R90H^* is strongly associated with SLE and possibly also with RA, but so far, no association has been found with PsA [7]. The current observation that the Ncf1-339 polymorphism, leading to an amino acid replacement NCF1^R90H^, is associated with mouse psoriasis, therefore calls for a deeper understanding of NOX2-mediated mechanisms through interactions with NOS2, which is likely of importance for the development of psoriasis.

Our study is based on the unique discovery that mannan exposure could induce the development of a disease in mice with the human Ncf1 polymorphism, mimicking PsO and PsA. Most importantly and uniquely, the MIP model is associated with the same major loci in the nonclassical MHC region (PSORS1) as the human disease [17]. Together with its characteristics, this makes the MIP model suitable not only for validation of new therapies but also for fundamental studies on the aetiology and pathogenesis of psoriasis.

## 5. Conclusions

We show that the human Ncf1-339 polymorphism (NCF1^90H^ variant) is associated with mouse psoriasis, confirmed by mannan-induced PsA and PsO mouse models. The NCF1^90H^ variant aggravates mouse psoriasis through hyperactivated macrophages with high NCF1 expression, expanded keratinocytes, upregulated IL-23/IL-17 axis, and JAK-STAT signaling pathway.

## Data Availability

All of the data is contained within the article and the Supplementary Materials.

## Supplementary Materials

The following supporting information can be downloaded at: https://www.mdpi.com/article/10.3390/antiox12071348/s1, Figure S1: Construction of Ncf1^90H^ mice; Figure S2: Flow cytometry gating strategy of immune cells and intracellular ROS; Figure S3: Flow cytometry gating strategy of immune cells in the spleen; Figure S4: The ratio of neutrophils and monocytes in the spleen; Figure S5: IHC staining in joint tissues; Figure S6: Flow cytometry gating strategy of γδT17 cells in mannan-induced mannan-induced PsA and PsO; Figure S7: Ratio of T cell subtypes in lymph nodes; Figure S8: Western blot of JAK2/STAT1 in skin tissues; Table S1: RT-qPCR primer sequences for mouse genes.
