# Supplementary material for: Human NCF190H Variant Promotes IL-23/IL-17—Dependent Mannan-Induced Psoriasis and Psoriatic Arthritis
Source: Antioxidants (Basel). 2023 Jun 27;12(7):1348. doi: 10.3390/antiox12071348 (PMC10376330; doi:10.3390/antiox12071348)

antioxidants

Supplementary Materials:

Table S1. RT-qPCR primer sequences for mouse genes.

Gene	Forward (5'–3')	Reverse (5'–3')	NCBI GeneID	GenBank Accession	Primer Bank ID
Ncf1	GTGGTCTACAGAAAATTCACCGA	CCATGAGGCCGTT-GAAGTATTC.	17969	NM_010876	170172552c2
Ncf2	TCACCAAGACAACTTTTCTGGC	GGCCCAGTTATCACTGCCC	17970	NM_010877	146134362c3
Ncf4	GTCATCGAGGTCAAAACAAAAGG	GCCCATGTAGACTTTGGCTG	17972	NM_008677	6679022a1
IL-17A	GGCCCTCAGACTACCTCAAC	TCTCGACCCTGAAAGTGAAGG	16171	NM_010552	142367609c2
IL-23	AATAATGTGCCCCGTATCCAGT	GCTCCCCTTTGAAGATGTCAG	83430	NM_031252	133892789c2
TNF-α	CCCTCACACTCAGATCATCTTCT	GCTACGACGTGGGCTACAG	21926	NM_013693	7305585a1
β-actin	GGCTGTATTCCCCTCCATCG	CCAGTTGGTAACAATGCCATGT	11461	NM_007393	6671509a1

Figure S1. Construction of *Ncf1^{90H}* mice. (A) Recombinant strategy to create the *Ncf1^{90H}* mutation in mice. (B) Agarose gel electrophoresis for *in vitro* transcribed Cas9 mRNA and guideRNA. (C) PCR products were separated by gel electrophoresis on a 1% agarose gel. (D) The PCR product used for sequencing and Seq peak data identifying homozygous, heterozygous, and wild type mice.

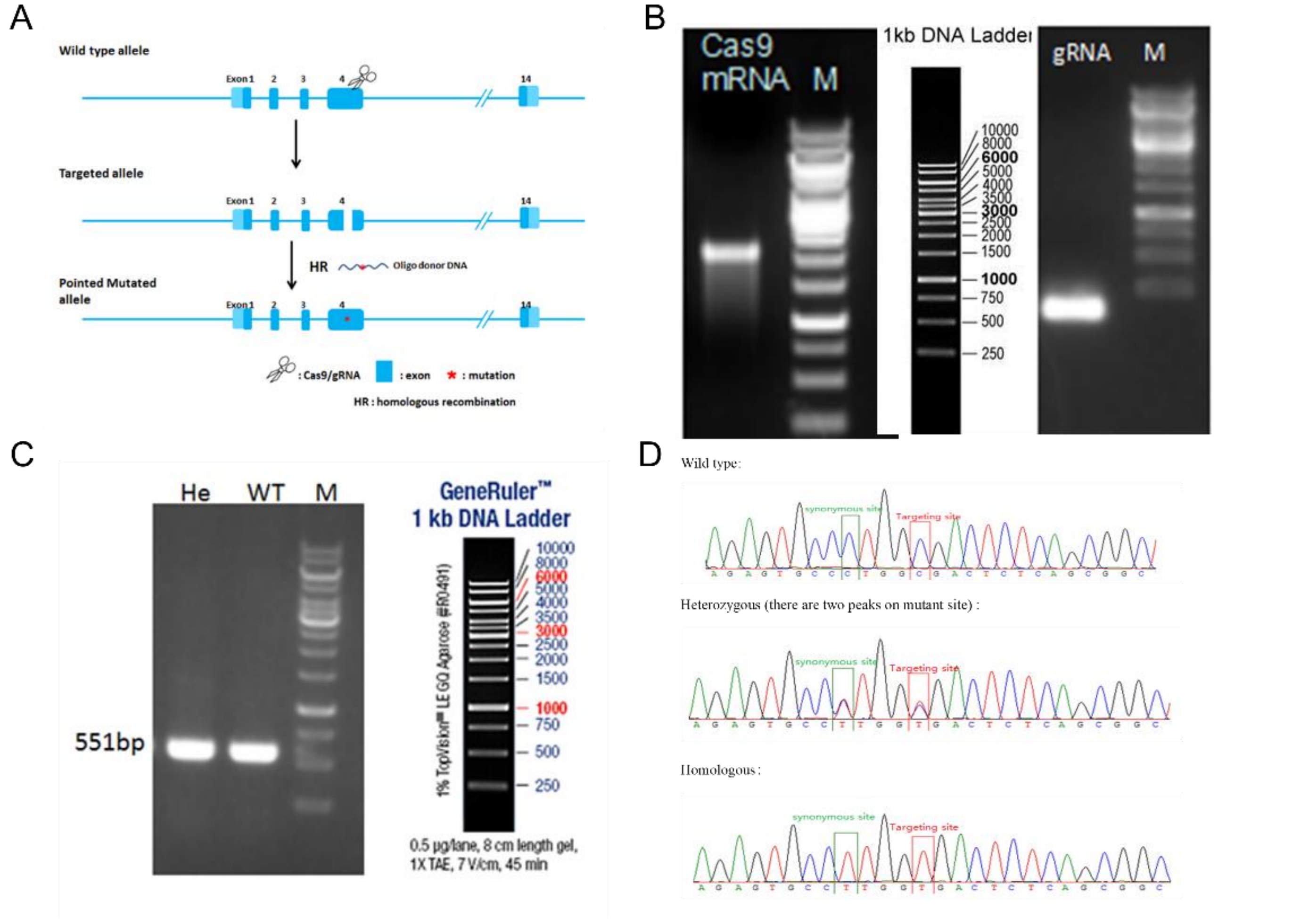

Figure S2. Flow cytometry gating strategy of immune cells and intracellular ROS. Monocytes, neutrophils, and macrophages, and ROS production in (A) peripheral blood, (B) bone marrow and (C) spleenrespectively.

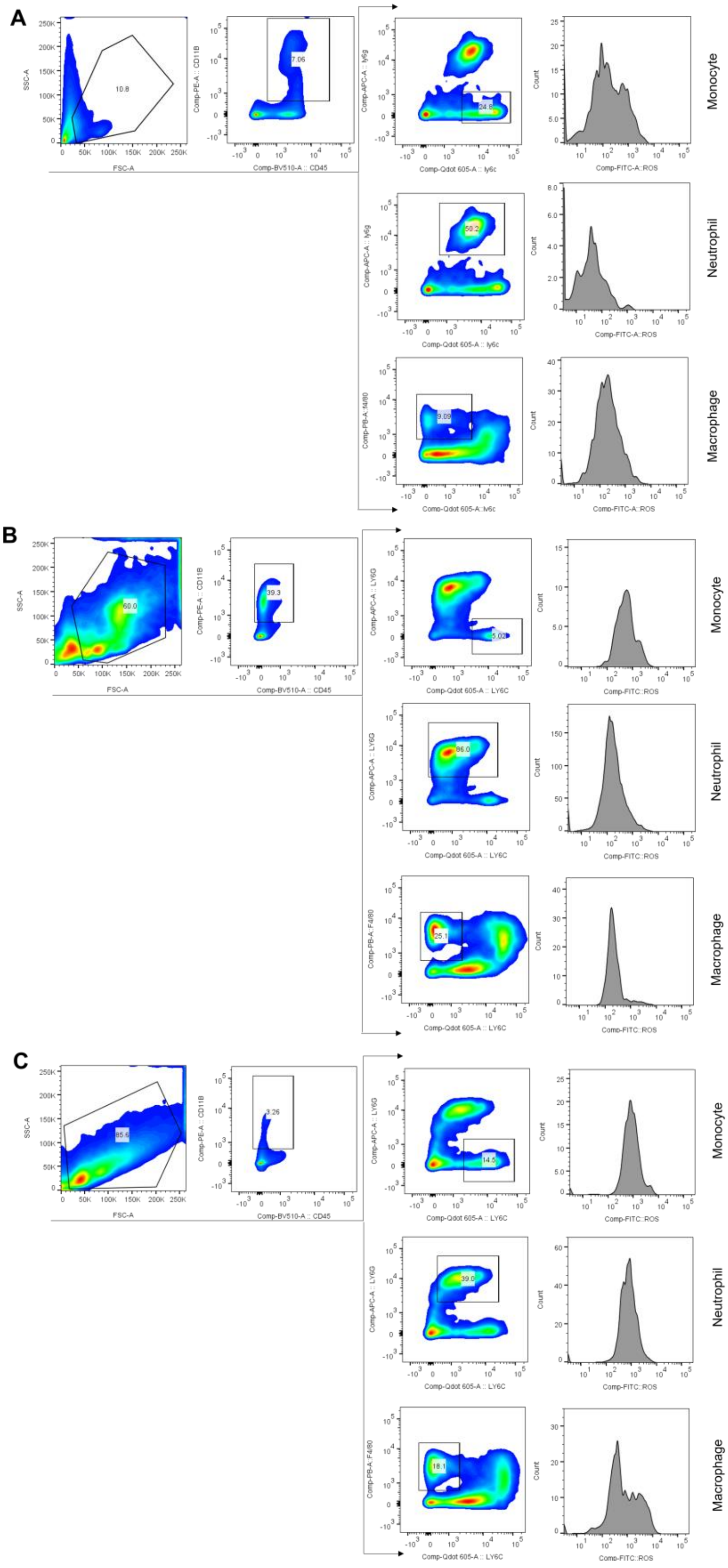

Figure S3. Flow cytometry gating strategy of immune cells in the spleen. (A) macrophages ($CD11b^{+}$ $F4/80^{+}$ $Ly6C^{-}$), and (B) pDCs ($CD45^{+}$ $CD11c^{-}$ $Ly6C^{+}$ $B220^{+}$ $PDCA1^{+}$).

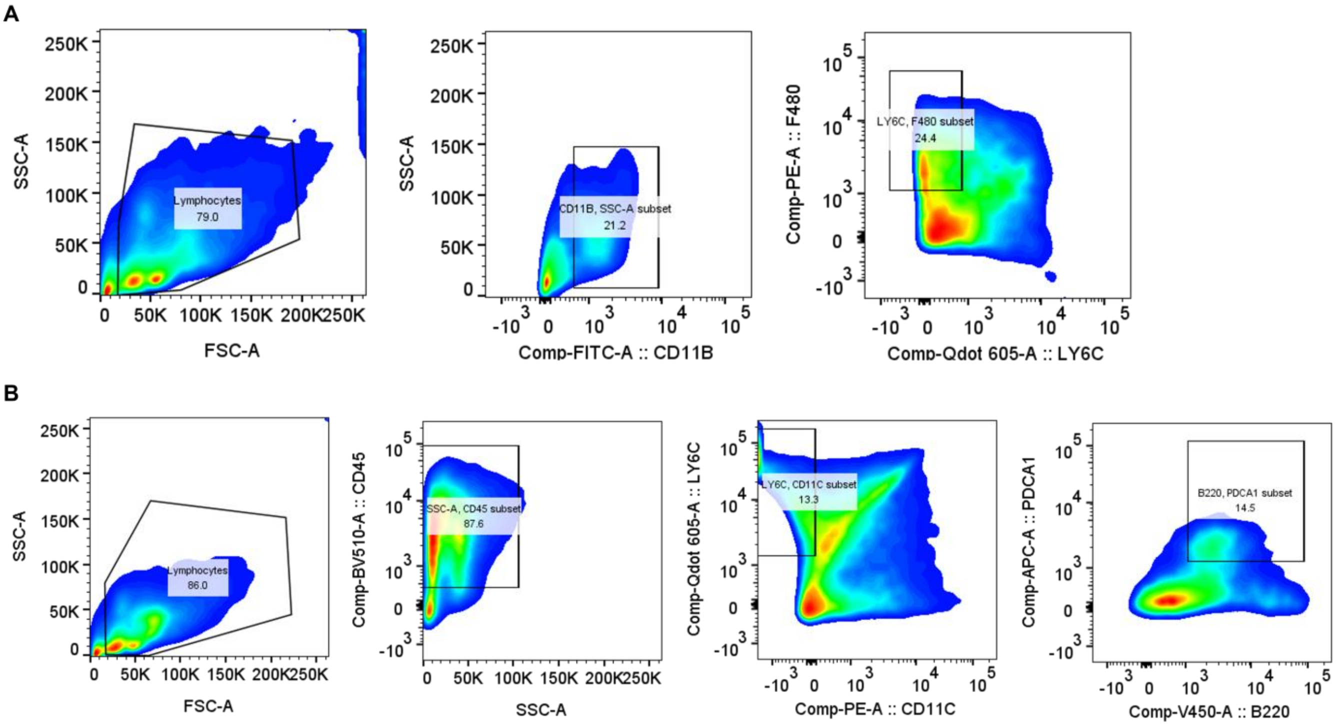

Figure S4. The ratio of neutrophils and monocytes in the spleen. (A) neutrophils ($CD11b^{+}$ $Ly6G^{+}$ $Ly6C^{int/-}$), and (B) monocytes ($CD11b^{+}$ $Ly6C^{+}$ $Ly6G^{-}$) in living splenocytes in mannan-induced psoriatic arthritis mice on day 10.

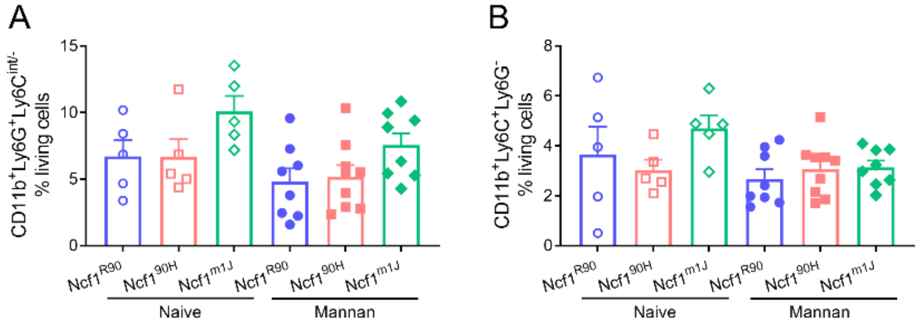

Figure S5. IHC staining in joint tissues. Macrophages (F4/80^{+}), NCF1 (p47phox) or p-STAT3 in the joint tissues from naïve and mannan-induced mice by intraperitoneal injection.

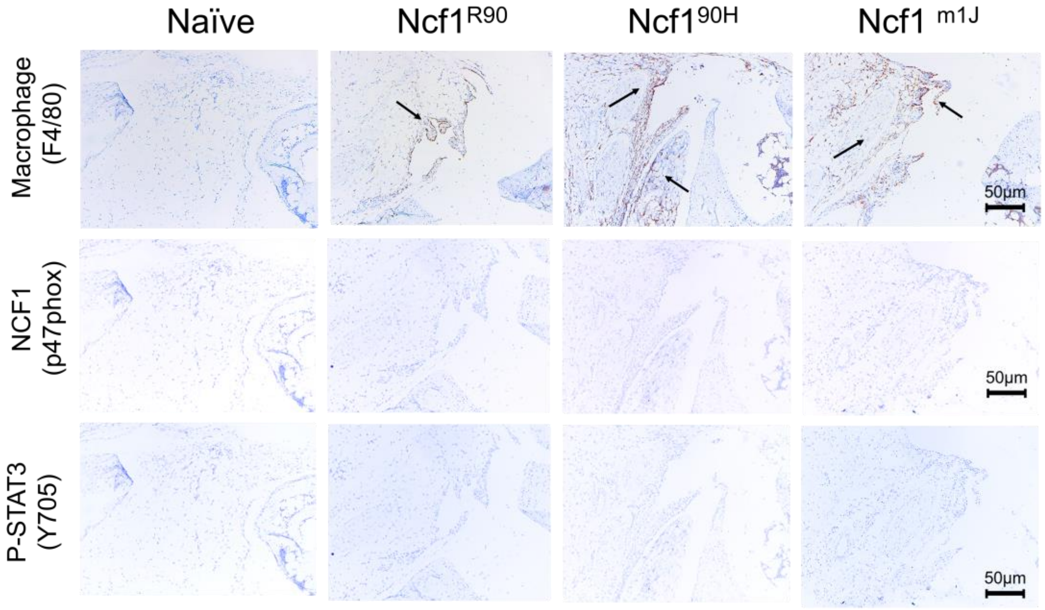

Figure S6. Flow cytometry gating strategy of γδT17 cells in mannan-induced mannan-induced PsA and PsO. (A) Intraperitoneal injection of mannan. (B) Epicutaneous application of mannan.

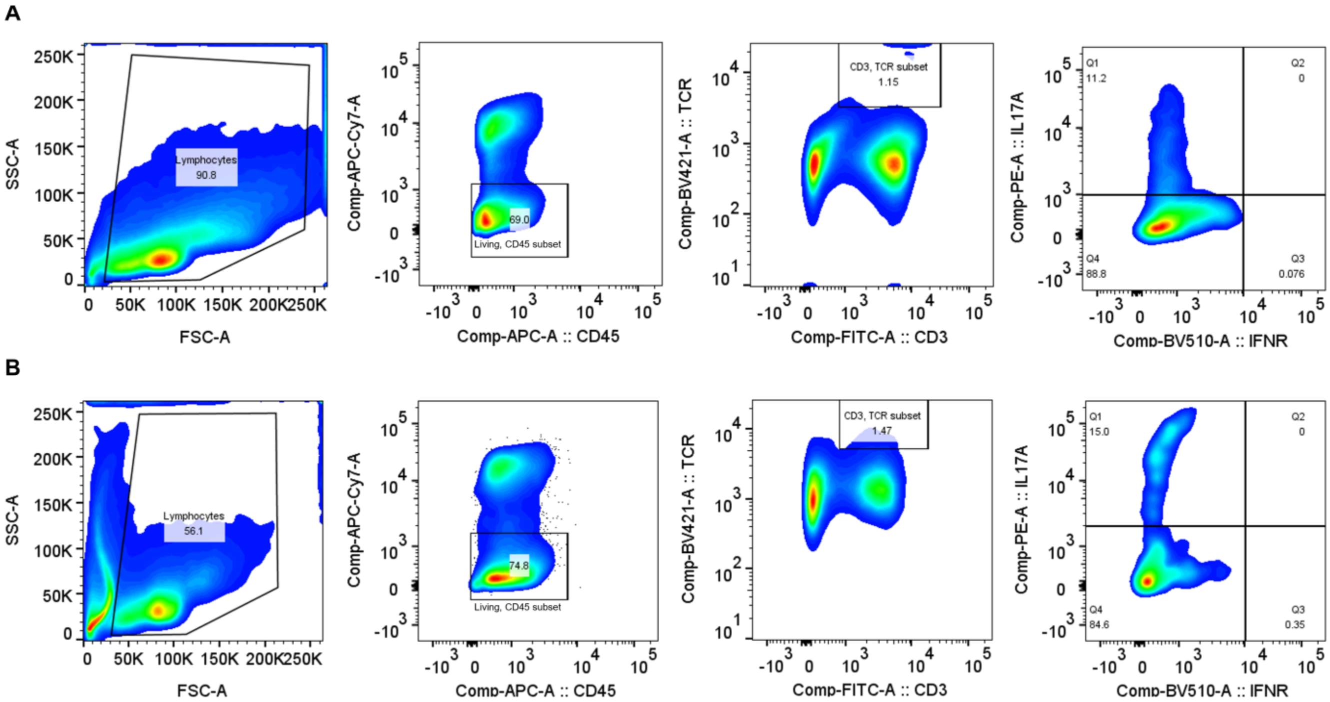

Figure S7. Ratio of T cell subtypes in lymph nodes. $CD3^+$ $CD4^+$ T cells in living $CD45^+$ cells, Th1 (TCR^+ IFN-γ^+) and Th17 (TCR^+ IL-17A^+) cells in $CD3^+$ $CD4^+$ T cells on day 10. (A) Intraperitoneal injection of mannan. (B) Epicutaneous application of mannan.

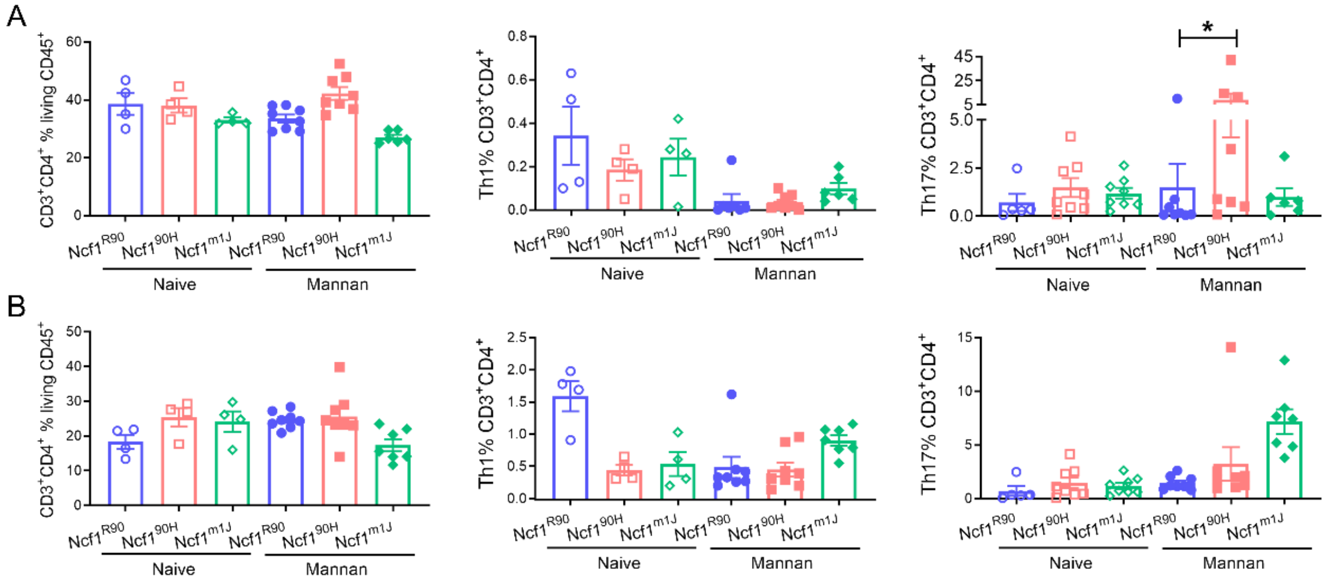

Figure S8. Western blot of JAK2/STAT1in skin tissues. *Ncf1^90H^* allele has no effect on JAK2/STAT1 signaling pathway in mannan-induced psoriatic skin tissues.

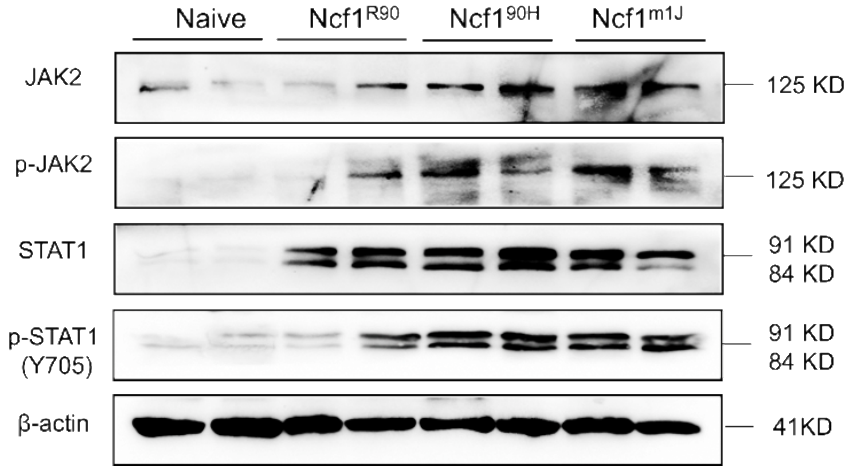

Supplement: Supplementary file 1 [file antioxidants-12-01348-s001.zip › antioxidants-2413946-supplementary.pdf]
